# Climate Change, Precipitation and Impacts on an Estuarine Refuge from Disease

**DOI:** 10.1371/journal.pone.0018849

**Published:** 2011-04-28

**Authors:** Jeffrey Levinton, Michael Doall, David Ralston, Adam Starke, Bassem Allam

**Affiliations:** 1 Department of Ecology and Evolution, Stony Brook University, Stony Brook, New York, United States of America; 2 Woods Hole Oceanographic Institution, Woods Hole, Massachusetts, United States of America; 3 School of Marine and Atmospheric Sciences, Stony Brook University, Stony Brook, New York, United States of America; Heriot-Watt University, United Kingdom

## Abstract

**Background:**

Oysters play important roles in estuarine ecosystems but have suffered recently due to overfishing, pollution, and habitat loss. A tradeoff between growth rate and disease prevalence as a function of salinity makes the estuarine salinity transition of special concern for oyster survival and restoration. Estuarine salinity varies with discharge, so increases or decreases in precipitation with climate change may shift regions of low salinity and disease refuge away from optimal oyster bottom habitat, negatively impacting reproduction and survival. Temperature is an additional factor for oyster survival, and recent temperature increases have increased vulnerability to disease in higher salinity regions.

**Methodology/Principal Findings:**

We examined growth, reproduction, and survival of oysters in the New York Harbor-Hudson River region, focusing on a low-salinity refuge in the estuary. Observations were during two years when rainfall was above average and comparable to projected future increases in precipitation in the region and a past period of about 15 years with high precipitation. We found a clear tradeoff between oyster growth and vulnerability to disease. Oysters survived well when exposed to intermediate salinities during two summers (2008, 2010) with moderate discharge conditions. However, increased precipitation and discharge in 2009 reduced salinities in the region with suitable benthic habitat, greatly increasing oyster mortality. To evaluate the estuarine conditions over longer periods, we applied a numerical model of the Hudson to simulate salinities over the past century. Model results suggest that much of the region with suitable benthic habitat that historically had been a low salinity refuge region may be vulnerable to higher mortality under projected increases in precipitation and discharge.

**Conclusions/Significance:**

Predicted increases in precipitation in the northeastern United States due to climate change may lower salinities past important thresholds for oyster survival in estuarine regions with appropriate substrate, potentially disrupting metapopulation dynamics and impeding oyster restoration efforts, especially in the Hudson estuary where a large basin constitutes an excellent refuge from disease.

## Introduction

Estuaries are biologically productive, supporting rich fisheries and diverse habitats, including oyster reefs, sea grass meadows, and vast expanses of fringing marshes. But their very richness coincides with human habitation, which has resulted in damage from pollution, overfishing and habitat destruction. Mid-Atlantic estuarine fisheries have severely declined from habitat alterations, pollution and overfishing [1], [2], [3]. For example, the loss of a key species, the eastern oyster *Crassostrea virginica*, has had significant effects on estuarine ecosystems of eastern and Gulf Coast North America [4], [5]. Oysters and other estuarine bivalves affect estuarine water quality by removing particles [4], [6] and influencing nitrogen cycling [7]. Oyster reefs also create three-dimensional benthic habitat that enhances diversity of other suspension feeders and offers important refuge from predators [5], [8].

Oyster growth and disease rates vary substantially along the estuarine salinity transition between fresh and marine waters. Oysters on the Atlantic and Gulf Coasts exposed to marine salinity are readily infected by two diseases. The parasite *Haplosporidium nelsoni*, or MSX, caused 90–95% mortality in the eastern oyster *Crassostrea virginica* in Delaware Bay in the 1950s [9]. MSX likely arrived in eastern North America from Japan, perhaps through an intermediate oyster host, and has spread to the eastern oyster [10] from Florida to Nova Scotia. The infection period is seasonal and disease can be reduced by moving oysters to lower salinities where survival of MSX is poor [9]. However, oyster growth and reproductive success decreases in lower salinities, and survival rates decrease below 5 psu [11]. Oysters have faster growth rates in higher salinities, but MSX infections decrease survival [9], [12], with a few exceptions of evolved resistance to the disease [13]. MSX infections occur in the mesohaline and polyhaline zones of estuaries, but infection rates are much lower and often absent and oysters can grow in oligohaline zones of 6–12 psu [9], [14].

A similar tradeoff between growth and disease exists for the other major oyster disease, the alveolate protistan *Perkinsus marinus* known as Dermo [15], [16], [17], [18]. First discovered on the Gulf Coast of the United States [19], it has spread to the northeast and is a major source of mortality in marine waters. Increases of coastal sea surface temperature over the past few decades [20], especially in the form of winter warming, have facilitated the disease's northward spread [21], [22]. Like MSX, Dermo does not thrive in oligohaline salinities [23], [24].

In oligohaline waters, oysters grow slowly but have refuge from disease and from marine predators like whelks, oyster drills, flatworms, and starfish. In watersheds with controlled discharge, experiments have suggested that periods of increased river flow can temporarily reduce oyster disease, with enhanced growth during subsequent lower discharge periods [25], [26]. In natural estuaries, seasonal and interannual variability in river discharge leads to continuous variation in salinity. High discharge during freshets will lower salinity at a location, but droughts will increase salinity and potentially increase disease susceptibility [27]. At the upper end of an estuary, increases in discharge may negatively impact oyster survival by reducing the frequency and duration of oligohaline conditions, making habitat that was formerly estuarine into a tidal freshwater river.

Oyster restoration is a priority in many estuaries of eastern North America, and in particular the Hudson River estuary [28]. The Hudson River estuary once supported among the richest oyster grounds in eastern North America [29], but signs of overfishing appeared early in the 19^th^ century, and urban pollution hastened the decline in the early 20^th^ century [29]. Jamaica Bay supported thousands of oyster fishers through the 19^th^ century [30], but oyster populations are now negligible there due to pollution, habitat disturbance and the 1938 hurricane. The Tappan Zee-Haverstraw Bay (TZ-HB) region is a focus of restoration efforts in the Hudson due to historic oyster cultivation in that part of the estuary. In the 18^th^ and 19^th^ centuries Haverstraw Bay supported commercial oyster fisheries [29]. In the 1950s, a time of below-average rainfall over the past century, the Flower and Sons Oyster Company moved their operation to the TZ-HB Bay region and raised juvenile oysters with high growth rates and survival [31]. These results raised hopes that the broad shallow waters of TZ-HB with suitable bottom substrate and high benthic population densities would be well suited for oyster restoration [32].

Oyster restoration objectives include not only reestablishment of fisheries, but also revitalization of a critical element of the estuarine ecosystem for increasing biodiversity and improving water quality. The current poor state of eastern oyster populations has led to skepticism for restoration potential [33], despite some successful efforts [34]. Climate change is a one potential challenge for restoration. Increased sea surface temperature has facilitated the northward extension of Dermo [22] and MSX [35], threatening oyster habitat in polyhaline and oceanic salinities. Regional shifts in timing and magnitude of precipitation with climate change will alter river discharges and estuarine salinities. Current climate models predict an increase in precipitation in the northeast U.S. of 5 to 8% in the next few decades and up to 30 percent by the end of the century with increases most likely in the winter and spring [36], [37], [38]. Climate projections also suggest greater variability in streamflow with more frequent high and low discharge periods [36], [37], [38]. The shifts in magnitude and timing of precipitation and discharge will affect the salinity distributions in estuaries and therefore the habitat, growth, and vulnerability of oyster populations and associated species. While estuarine oysters can tolerate freshwater during the winter, very low salinities cause high degrees of physiological stress under spring and summer temperature conditions [11].

We are examining the Hudson River estuary, once a major oyster grounds and now a focus for restoration. We have combined regional studies of oyster performance (growth rate and survival) and estuarine modeling to predict physical conditions and potential impacts on oyster habitat under a regime of increased precipitation with climate change.

## Results

**Figure 1:**
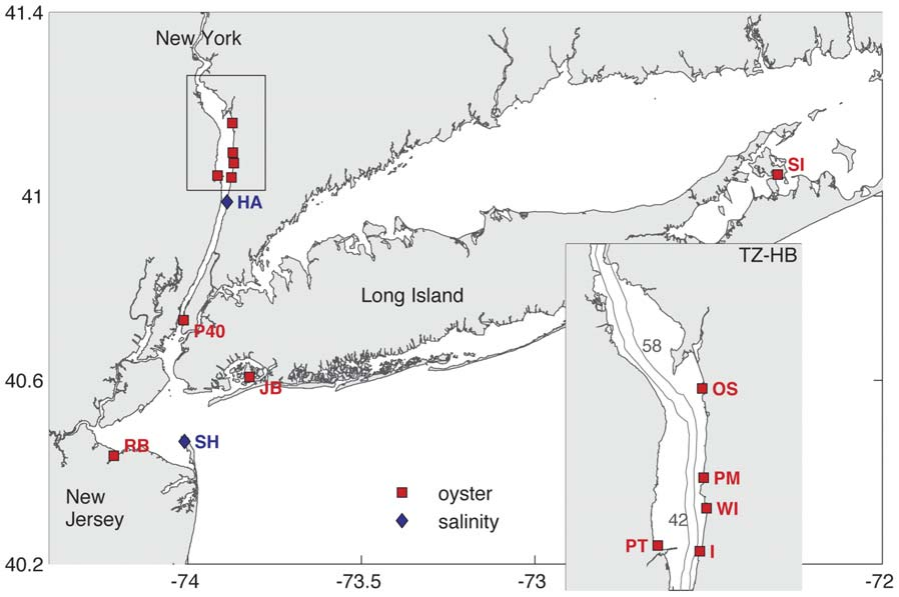
Map showing observation locations. Red squares are oyster test stations: OS = Ossining, PM = Philips Manor, PT = Piermont, WI = Tarrytown (Washington Irving Boat Club), I = Irvington, P40 = Pier 40, SI = Shelter Island, JB = Jamaica Bay, RB = Raritan Bay, New Jersey. Blue diamonds show salinity measurement locations (data in Figure 3b): HA = Hastings (USGS), SH = Sandy Hook (NOAA). The inset focuses on the Tappan Zee-Haverstraw Bay (TZ-HB) region; also noted for reference are the along estuary distances of 42 km and 58 km (from the Battery at the southern end of Manhattan) and 10-m isobath at the transition between the channel and shoals.

**Figure 2:**
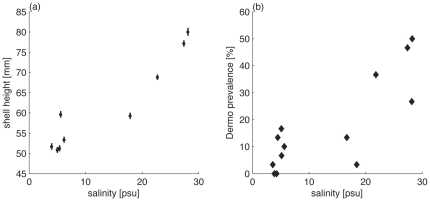
Growth and disease prevalence as a function of salinity. (a) Relationship of mean oyster shell height to salinity (r^2^ = 0.89, in samples collected in October 2008, after 3 months of growth from a mean starting height of 51.7 mm);vertical bars show standard error. (b) Prevalence of Dermo in oysters (30 per site) from 9 sites taken from coastal and TZ-HB sites in September, 2008, and 4 sites from coastal and TZ-HB sites in August, 2009 (r^2^ = 0.67).

We investigated oyster performance in coastal and estuarine regions to evaluate tradeoffs between performance and disease occurrence. We compared growth, survival, reproduction, and disease occurrence at coastal sites from eastern Long Island, New York USA to western Raritan Bay, New Jersey USA and at sites in the TZ-HB section of the Hudson River estuary (river km 42–58) (Figure 1). We quantified shell growth and disease prevalence (Dermo) of overwintered oysters that were transplanted from one hatchery (Fishers Island, New York USA) to replicate floating cages at 9 sites in 2008, and to 5 of these sites in 2009 (Figure 2, see “Materials and Methods”). Shell growth showed a strong positive correlation with salinity (Figure 2). In contrast, Dermo was far less prevalent in the lower salinity sites of the estuary than in coastal sites. These results are consistent with the expected tradeoff between growth and disease with salinity, documenting the tradeoff more completely and at higher latitudes than previous work.

The prevalence of MSX was low at most sites during 2008 and 2009. MSX was responsible for substantial mortality in 2008 at one site in the lower Hudson estuary (Pier 40, “P40” in Figure 1, with a mean cumulative mortality of 43%). This elevated mortality due to MSX occurred near the mouth of the estuary, a location with higher salinities and greater salinity variability than the upper estuary sites in TZ-HB.

**Figure 3:**
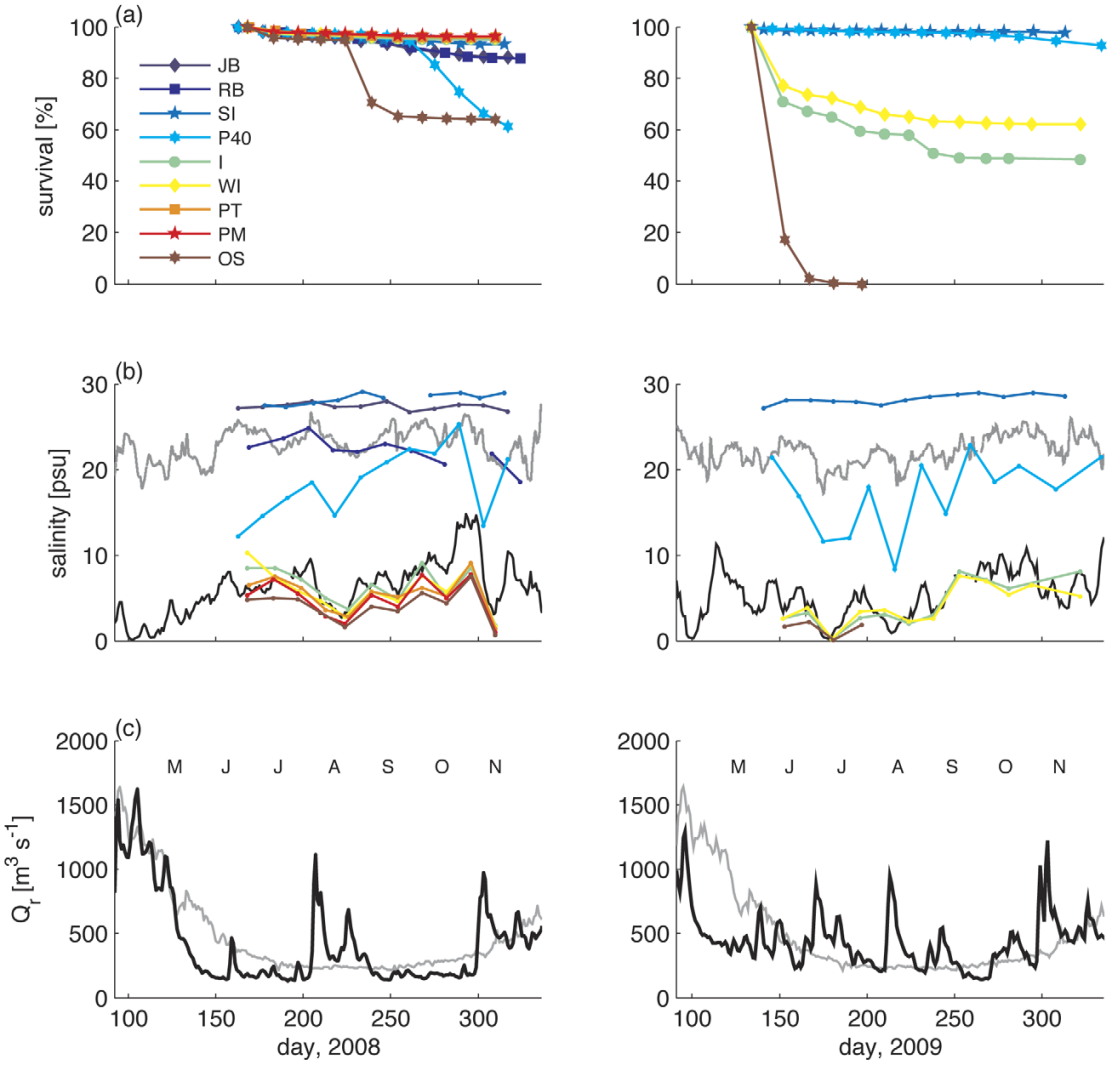
Survival patterns, salinity variation, and river discharge. (a) Left: Survivorship of oysters grown in summer 2008 at a series of coastal and oligohaline sites in Tappan Zee-Haverstraw Bay. The decline at Ossining, the lowest-salinity TZ-HB site, was associated with a drop of salinity while the decline at Pier 40 was associated with a major infection of MSX. Right: Survivorship of oysters grown in the summer of 2009 (only 5 of the 2008 sites were investigated), comparing TZ-HB with two of the coastal sites studied in 2008. (b) Salinities in 2008 (left) and 2009 (right). Continuous, tidally filtered surface salinities are shown for Sandy Hook NJ (NOAA station # 8531680, SH in Figure 1) and Hastings NY (USGS station # 01376304, HA in Figure 1) (grey and black lines, respectively); oyster sites were sampled biweekly. (c) River discharge in 2008 (left, dark line) and 2009 (right), as compared to average seasonal discharge pattern for the period 1918–2009 (light grey line).

Both 2008 and 2009 had higher than average precipitation (measured at Albany) and discharge in the Hudson River, but the timing of the high discharge period appears to be critical. At the TZ-HB sites salinities were in the range of 5–10 psu through July and August of 2008. We found generally high survival rates (Figure 3a), albeit with low growth rates (Figure 2b). During a discharge event in August 2008 salinity decreased below 2 psu at the site farthest up-estuary (Ossining, “OS” in Figure 1), corresponding with a mortality increase of ca. 30 percent.

Precipitation and river discharge during the summer months of 2009 were greater than in 2008, with lower salinities at the estuarine stations and much greater mortality in TZ-HB (Figure 3b). In contrast, mortality was minimal at the coastal sites in the study. In 2009, salinities in TZ-HB dropped to nearly 0 and remained around 3 psu for most of the summer. The populations farthest up-estuary (“OS”) died off completely and two other TZ-HB sites (“I” and “WI”) had significant mortality. Mortality at “I” and “WI” decreased in October as river discharge declined and salinity increased.

A limited extension of the observations into 2010 offers additional evidence of the sensitivity of oyster growth and survival in TZ-HB to summer river discharge. Soft tissue growth, shell height growth, and survival were measured at the Washington Irving Boat Club in Tarrytown (“WI”) during the summers of 2008, 2009 and 2010 (Table 1). In 2009, summer precipitation and discharge were high: precipitation at Albany (averaged May 1 to September 1) was the highest in the 132 year record and average discharge in the Hudson ranked 13^th^ in the 93 year record. Average summer precipitation and discharge in 2008 and 2010 were significantly lower. Correspondingly, oyster growth at the Tarrytown site was much less and mortality was greater during the wet summer of 2009 than 2008 or 2010.

**Table 1:** Soft tissue growth (g), shell height growth (cm), and survival (percent), relative to salinity during the growing season (numerical model estimates for June 1–September 22 of 2008, 2009, and 2010) at the Washington Irving Boat Club in Tarrytown (“WI” in Figure 1).

Year	Tissue±S.E. (N)	Height±S.E. (N)	Survival (%)	Days over 5 psu
2008	0.40±0.03(40)	8.62±0.81(80)	95.9	61
2009	0.14±0.01(40)	4.71±0.56(80)	62.3	6
2010	0.49±0.04(20)	24.02±0.79(60)	87.9	83

To relate conditions during the observations to longer-term estuarine variability, we use a numerical model of the circulation and salinity in the Hudson River estuary that has previously been validated against observations [39], [40]. A hindcast of the salinity variability over the past ca. 90 years was made using the available discharge and tidal records. The model calculates the vertical salinity structure as well as the along-estuary distribution, and here we focus on salinities in the relatively shallow regions (depths less than about 3 m) on the east side of TZ-HB where leases for oyster culture were maintained in the 1950s and where restoration is most likely [40]. Estuarine salinity depends inversely on discharge – as discharge increases, salt is pushed toward the mouth and salinity decreases.

**Figure 4:**
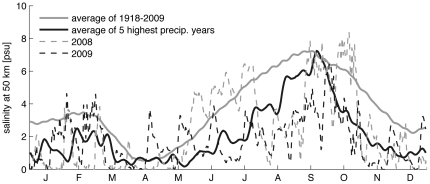
Model simulations of salinity at TZ-HB site (river km 50). Shown are average conditions over the entire period 1918–2009, and average conditions during the 5 years of that period with the greatest annual precipitation. Model output is averaged by year-day and filtered with a 5-day running average, Daily average salinities from the model at the same location are shown for 2008 and 2009.

The model suggests that during high discharge periods, salinities in TZ-HB are frequently low enough to limit oyster growth and even survival (Figure 4). For example, averaging over the 90-year record, salinities in summer months in TZ-HB were in the range of 3 to 7 psu. In contrast, average salinities during the 5 years with the highest annual precipitation were on average 2 to 3 psu lower during the summer months. Model results in TZ-HB are also shown for 2008 and 2009. The increased precipitation during the late spring and summer of 2009 lead to decreased salinities during the summer, with salinities similar to the average conditions during historically high discharge years. While the annual precipitation and discharge were greater in 2008 than in 2009, the high discharge period in 2008 was during the typical spring freshet rather than during the summer months of oyster recruitment.

**Figure 5:**
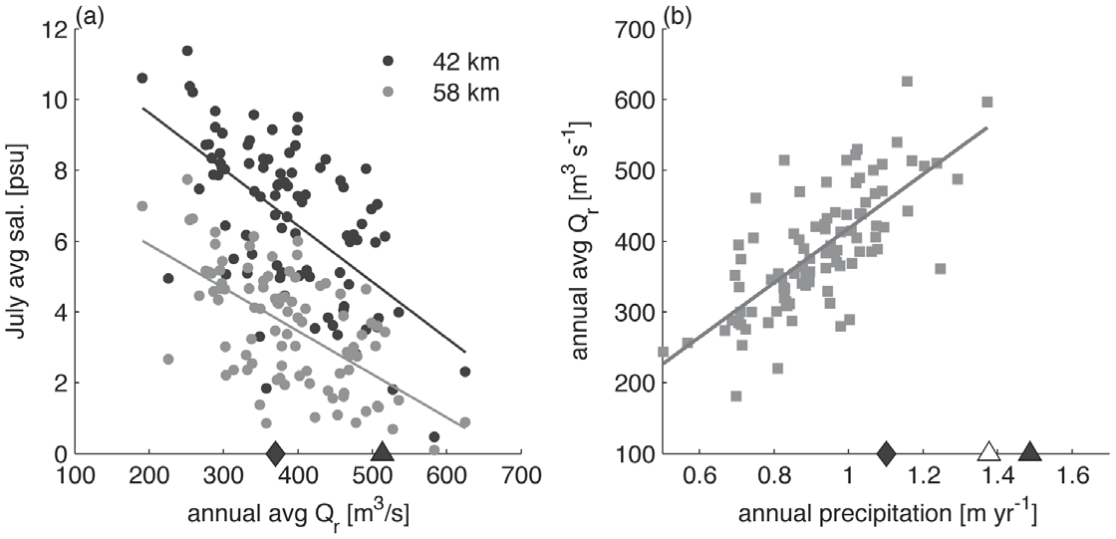
(a) Average salinity in July in lower (42 km) and upper (58 km) TZ-HB from model results against average discharge for the water year. Analysis of covariance shows slopes to be not distinguishable in value over data from 90 years (F = 1.89, p∼0.17) but trend lines are significantly displaced (F = 175.23, p<0.001). The diamond marker indicates the median discharge over the period, the closed triangle corresponds with discharge averaged for five wettest years. (b) Relationship between annual rainfall at Albany NY and annual river discharge (r^2^ = 0.57). The diamond and closed triangle markers are as in (a). The open triangle is a crude projection of the precipitation with climate change (25% increase).

Salinities from upper (river km 58) and lower (river km 42) TZ-HB over the full historical simulation demonstrate the inverse dependence between summer salinity (average July salinity shown here) and mean annual discharge (Figure 5a). Conditions in the upper bay range from about 6 psu to essentially fresh, while the lower bay ranges from about 10 to less than 3 psu. The mean annual discharge and the mean discharge of the 5 years with greatest precipitation are indicated with markers on the abscissa for reference. The model results indicate that during high discharge years, only a very limited region of TZ-HB would retain sufficiently high salinities in summer to provide suitable oyster habitat.

Mean annual discharge in the Hudson River depends primarily on regional precipitation (Figure 5b). Most current climate models project increases in precipitation in the U.S. Northeast in the coming decades, with the greatest increases during winter and spring [36]. Climate models predict a range of outcomes for summer precipitation [36], [37]. Oyster restoration prospects are sensitive to these projections, as summer is the season of oyster larval recruitment. The total projected increase in precipitation in the Northeast over the coming century is about 25 percent, similar to the difference between the average annual precipitation over the past 90 years and the average of the 5 highest precipitation years. If predictions of increased precipitation hold, particularly during the summer months, then decreases in salinity in TZ-HB may be detrimental to oyster survival and therefore restoration.

In addition to the local salinity, the rate of salinity change can be a source of physiological stress [11] and is a factor in oyster restoration. During high discharge, the estuarine salinity distribution compresses, and variability in salinity from tidal and meteorological forcing increases at any particular point due to the sharper salinity gradient. The temporal variability in salinity would be exacerbated by projected increases in the intensity of extreme precipitation events with climate change [36], [37]. Overall, the projected increase in precipitation and discharge would be expected to shift the location of suitable habitat for oyster growth. The bathymetry of the Hudson is such that appropriate water depths and appropriate substrate for oyster growth are sparse down-estuary from TZ-HB, so the total area with suitable water column and benthic conditions could be expected to decrease with a shift in the salinity distribution toward the mouth. Within the TZ-HB region, extensive suitable bottom areas exist that would support oyster growth and widespread larval recruitment has been observed there [41].An additional consideration is that increases in water temperature may exacerbate negative impacts of disease in oysters, particularly at coastal sites.

## Discussion

At present, the Tappan Zee – Haverstraw Bay region of the Hudson estuary provides suitable benthic habitat for oysters and a likely refuge from Dermo and MSX diseases. However, increased mortality in TZ-HB during the high discharge summer months of 2009 suggest that projected increases in precipitation with climate change may reduce salinities in this region below thresholds for oyster survival. Our modeling results suggest that discharges consistent with precipitation in future climate scenarios could decrease salinities in the region to levels below the threshold for oyster survival. The seasonal timing of precipitation and discharge remains a critical uncertainty in this assessment. While climate models generally agree that precipitation is likely to increase during winter and spring in the Northeast [36], [37], uncertainty remains for the summer months that are important for oyster growth, spawning, and larval dispersal. Historically, high annual average precipitation correlates with lower salinities in July due to longer, higher volume freshets (Figure 5a). Whether the trend continues depends on the future partitioning of precipitation between snow and rain and its effect on the timing of river discharge. Independent of the seasonal distribution, projections of increased variability in streamflow [36], [37] are likely to be a stressor to oyster communities at the upstream margins of estuaries.

Restoration of oyster populations in TZ-HB could have important implications for oysters throughout the Hudson-Raritan region. If populations could be restored, larvae from TZ-HB Bay might be exported to coastal sites in years when coastal populations with higher vulnerability to disease and predators fail to reproduce or survive. We found oysters recruiting to our cages in TZ-HB in the late summer of 2008, but could not determine if the larvae came from within the bay or from down estuary. Our observations in 2009 showing no recruitment in Jamaica Bay or the New York Harbor region suggests that the recruitment within TZ-H may have been indigenous. Thus the possibility for a metapopulation of interacting disease-prone, but high growth rate oysters on the coasts and low growth rate but disease-free oysters in the TZ-HB region could provide temporal reinforcement and promote overall survival of the regional oyster metapopulation [42]. A model of connectivity has not yet been developed for this region, but restoration efforts would depend on maintaining a metapopulation of rapidly growing and disease-resistant local populations. In Chesapeake Bay, connections of similar distances have been shown to be feasible according to modeling studies [43].

A broader assessment of effects of regional precipitation shifts on oyster populations in estuaries in eastern North America and the Gulf Coast could relate results to metapopulation design to maximize oyster recruitment and survival [44]. Salinity structure in Chesapeake Bay, for example, is driven by variation of discharge in the major tributaries, particularly the Susquehanna [36]. Anticipated increases in precipitation from climate change may cause major losses of oysters and estuarine habitat as salinity decreases, particularly in tributaries in the middle of the bay where isohalines may shift seaward by as much as 55 km [36]. Delaware Bay has a small watershed and increased rainfall might have a salutary effect, driving low salinity waters and disease refuge into the shallow bay. Previous droughts were associated with expanded mortality from MSX as saline water moved into the upper reaches of Delaware and Chesapeake Bays [9], [45]. In general, the impacts of climate change on estuarine oyster populations will depend on how the modified salinity distribution corresponds to the location of suitable benthic habitat. The uncertainty of seasonal effects on changes in rainfall [36] will strongly affect our predictions of potential for oyster restoration.

The summer of 2009 was notable for increased precipitation and discharge during the late spring and summer, but climate predictions suggest increased precipitation may become more common in the future. In the Hudson, the shoals of Tappan Zee and Haverstraw Bay may evolve from a refuge from disease to an inhospitable habitat for oysters, eliminating a crucial component of a larger metapopulation. Even a decade of rainy years, such as the past decade in the Hudson, could hinder restoration efforts. Oyster restoration planning should take into consideration the response of the oligohaline transition between estuarine and fresh waters to potential shifts in forcing with climate change, in particular the magnitude and seasonal timing of discharge. The resilience of restored estuarine oysters may depend on the availability and proximity of suitable benthic substrate for colonization with shifts in the salinity regime. Significant uncertainty remains among predictions of climate change impacts on precipitation, as well as for other potential factors in oyster survival such as water temperature and sea level rise. Restoration efforts could address this uncertainty by focusing on estuarine regions that would allow for translation of the oysters in response to shifts in forcing and by continuously monitoring environmental conditions and oyster population response to better inform subsequent restoration efforts.

Similar effects of climate change on the spread of disease have been widely noted [46] and may portend major reorganization of natural communities in future decades. In the Hudson, the transitional zone of Tappan Zee-Haverstraw Bay and its vulnerability may provide lessons for estuaries throughout the world. The simultaneous effects of climate change on disease and physiological adaptations may give insight to the effect of regional climate change in other transitional environments.

## Materials and Methods

Eastern oysters, *Crassostrea virginica*, were placed in plastic mesh grow-out bags (14 mm mesh size) supported in wire cages suspended 1–2 meters below the surface at nine sites throughout the coastal New York, New Jersey, and Tappan Zee-Haverstraw Bay region (Figure 1, Table 2). Two semi-rigid, rectangular shaped (dimensions of 94×43×7.6 cm) grow-out bags were placed in each wire cage. 300 oysters were placed in each grow-out bag, resulting in a starting density of 742 oysters m^−2^. Oysters were purchased from the Fishers Island Oyster Farm and were spawned and settled in the summer of 2007 (data for Figure 2a) and overwintered before being transferred to the cages in June 2008. Oysters used in cages in 2009 were spawned and settled in the summer of 2008, overwintered and placed in cages in late May 2009 (data for Figure 2b, 1b). In coastal sites, three replicate cages (6 grow-out bags) were used, located about one meter apart. At Tappan Zee-Haverstraw Bay sites, two cages (4 grow-out bags) were each maintained one meter or more from the other. In both years, oyster height was measured with a random sample of 20 oysters from each sample bag without replacement every two weeks from June-November. We report oyster shell height for the October sampling. Since shell size was the same for all starting samples, the final mean shell height for a locality is a measure of shell growth. All live and dead oysters were counted to calculate survivorship. Cages and bags were cleaned of fouling organisms once every 2 weeks when measurements were taken.

**Table 2:** Localities, keys to localities, and GPS coordinates.

Locality Key	Site Description	GPS Location
OS	Ossining, Westerly Marina, Bulkhead	N 41°09.521′W 073°52.321′
PM	Philips Manor, Beach Club, floating dock	N 41°05.648′W 073°52.240′
PT	Piermont, Cornetta Marina	N 41°02.708′W 073°54.884′
WI	Tarrytown, Washington Irving Boat Club, permanent dock	N 41°04.320′W 073°52.077′
I	Irvington, Irvington Boat and Kayak Club, floating dock	N 41°02.463′W 073°52.450′
P40	Pier 40, cages suspended over the south side of the vessel *Lilac*	N 40°43.835′W 074°00.776
SI	Shelter Island, Log Cabin Creek in Mashomack Preserve, floating dock	N 41°02.832′W 072°18.020′
JB	Jamaica Bay, residence in Broad Channel, NY, floating dock	N 40°36.414′W 073°49.302
RB	Raritan Bay, New Jersey, Brown's Point Marina, floating dock	N 40°26.147′W 074°12.786′

Temperature was monitored with in situ temperature loggers (TidbiT v2 temp loggers from Onset Corporation) attached to one cage at each of the 9 localities. Temperature was registered every 15 minutes. Salinity, temperature and dissolved oxygen were measured biweekly at cage depth using a YSI model 85 environmental TSO meter.

Disease was assessed for occurrence and intensity of occurrence of MSX and Dermo in the laboratory. A sample of 30 oysters was tested once a year at each site in September. Oysters were dissected and biopsies of mantle and rectum tissues were incubated in Ray's fluid thioglycollate medium (RFTM) for the detection of *P. marinus* [19]. Following incubation (1 week), biopsies were stained with Lugol's iodine and examined using a light microscope for the presence of enlarged, black stained parasite cells. Infection intensity was ranked (0–5) following a scale assessing the relative abundance of parasite cells in tissues (0: no infection, 5:heavy infection) [47]. MSX detection was performed using standard histopathology procedures. Briefly, a transverse slice of tissue roughly between 3 and 5 mm in thickness was made through the central region of the visceral mass to include digestive organs, gonads, as well as gill and mantle tissues. Tissue sections were placed in histo-cassettes and fixed in 10% buffered formalin. Following fixation, tissue samples were dehydrated and embedded in paraffin, sectioned (5 to 6 µm in thickness), and mounted on histology slides. MSX infection intensity was ranked as light, moderate or heavy based on the abundance of parasite cells in tissue sections and following general guidelines [48].

The numerical model is an unsteady, quasi-2d solution for the along-estuary velocity and salinity distributions. The model has been previously applied to and validated for the Hudson River estuary based on comparisons with high resolution observations in a single year [45] and against observations over several decades, corresponding with simulations presented here [44]. The model was forced with river discharge upstream (USGS station #01358000 from 1946 to present, #01357500 from 1917, and #01335754, from 1887) and with tidal water level downstream (NOAA stations #8518750, #8531680, and #8534720). The model calculates the vertical structure of velocity and salinity at discrete points along the thalweg of the estuary (dx = 1 km). We extract model salinities at depths corresponding to the bed elevation on the shoals where the oyster sites were located. Precipitation observations were taken from Albany, NY (NCDC WBANID #14735 and #14796).

Work on oysters was done with permission under permits to the New York-New Jersey Baykeeper Oyster Gardener Program (Raritan Bay and Jamaica Bay) and, for the other sites, under New York State Fish and Wildlife Scientific Collecting License number 1257 to Jeffrey Levinton.
